# Vermessung der Cochlea mittels eines Tablet-basierten Softwarepakets: Einfluss der Bildgebungsmodalität und des Untersucherhintergrunds

**DOI:** 10.1007/s00106-022-01208-3

**Published:** 2022-08-15

**Authors:** Lena Weber, Pingling Kwok, Erin M. Picou, Christina Wendl, Christopher Bohr, Steven C. Marcrum

**Affiliations:** 1 Klinik und Poliklinik für Hals-Nasen-Ohren-Heilkunde, Universitätsklinikum Regensburg, Franz-Josef-Strauß-Allee 11, 93053 Regensburg, Deutschland; 2 Department of Hearing and Speech Sciences, Vanderbilt University Medical Center, 2201 West End Avenue, Nashville, TN 37235 USA; 3 Institut für Röntgendiagnostik, Universitätsklinikum Regensburg, Franz-Josef-Strauß Allee 11, 93053 Regensburg, Deutschland

**Keywords:** Cochleaimplantat, Länge des Ductus cochlearis, OTOPLAN, Magnetresonanztomographie, Computertomographie, Cochlear implant, Cochlear duct length, OTOPLAN, Magnetic resonance imaging, Computed tomography

## Abstract

**Hintergrund:**

Die Größe der Cochlea ist individuell unterschiedlich, was sich in der variablen Länge des Ductus cochlearis (CDL) ausdrücken lässt. In der Cochleaimplantatversorgung ist ein in der Länge angepasster Elektrodenträger durch eine optimale Abdeckung der Cochlea erfolgversprechend. Dazu kann die CDL auf Höhe des Corti-Organs (CDL_OC_) mittels eines Tablet-basierten Softwarepakets ausgemessen werden, um abgestimmt auf die Anatomie einen passenden Elektrodenträger auszuwählen.

**Fragestellung:**

Haben die Modalität der Bildgebung und der Untersucherhintergrund einen Einfluss auf die Vermessung der CDL?

**Methoden:**

Die Datensätze der Magnetresonanztomographie (MRT) und Flachdetektor-Volumen-Computertomographie (fpVCT) von 10 Patienten (20 Cochleae) wurden in der Software OTOPLAN (MED-EL, Innsbruck, Österreich) analysiert. Als Untersucher wurden eine Oberärztin der Hals-Nasen-Ohren-Heilkunde, eine Assistenzärztin der HNO-Heilkunde und ein Audiologe ausgewählt. Zur Analyse der Effekte der Bildgebung und des Untersucherhintergrunds auf die CDL-Messungen wurden linear gemischte Modelle konstruiert.

**Ergebnisse:**

Die Messungen ergaben einen Mittelwert CDL_OC_(fpVCT) = 36,69 ± 1,78 mm und CDL_OC_(MRT) = 36,81 ± 1,87 mm. Die Analysen zeigten keinen signifikanten Effekt des Untersucherhintergrunds auf die Messergebnisse (F (2, 105) = 0,84; *p* = 0,437). Die Bildgebungsmodalität zeigte einen signifikanten Einfluss (F (1, 105) = 20,70; *p* < 0,001), wobei die Messungen an MRT im Mittel um 0,89 mm größer waren.

**Schlussfolgerung:**

Da der Untersucherhintergrund keinen Einfluss auf die Messungen hatte, lässt sich schließen, dass die Messungen nicht ausschließlich von ärztlichem Personal, insbesondere nicht nur von erfahrenen Neurootologen, durchgeführt werden müssen. Die Methode der Bildgebung (fpVCT vs. MRT) kann die CDL-Werte statistisch signifikant beeinflussen, wobei eine klinische Relevanz fraglich ist.

## Abstract (en)

**Background:**

Cochlear duct length (CDL) is subject to significant individual variation. In the context of cochlear implantation, adapting the electrode array length to the CDL is of potential interest, as it has been associated with improvements in both speech recognition and sound quality. Using a tablet-based software package, it is possible to measure CDL at the level of the organ of Corti (CDL_OC_) to select appropriate electrode array lengths based on individual cochlear anatomy.

**Objective:**

To identify effects of imaging modality and rater background on CDL estimates.

**Methods:**

Magnetic resonance imaging (MRI) and flat-panel volume CT (fpVCT) scans of 10 patients (20 cochleae) were analyzed using the OTOPLAN software package (MED-EL, Innsbruck, Austria). Raters were an otorhinolaryngology (ORL) specialist, an ORL resident, and an audiologist. To analyze effects of rater background and imaging modality on CDL measurements, linear mixed models were constructed.

**Results:**

Measurements showed mean CDL_OC_(fpVCT) = 36.69 ± 1.78 mm and CDL_OC_(MRI) = 36.81 ± 1.87 mm. Analyses indicated no significant effect of rater background (F(2, 105) = 0.84; *p* = 0.437) on CDL estimates. Imaging modality, on the other hand, significantly affected CDL (F (1, 105) = 20.70; *p* < 0.001), whereby estimates obtained using MRI were 0.89 mm larger than those obtained using fpVCT.

**Conclusion:**

No effect of rater background on CDL estimates could be identified, suggesting that comparable measurements could be obtained by personnel other than specially trained neurootologists. While imaging modality (fpVCT vs. MRI) did impact CDL results, the difference was small and of questionable clinical significance.

## Hintergrund

Cochleaimplantate stellen für Patienten mit Taubheit und hochgradiger Schwerhörigkeit eine etablierte Form der Hörrehabilitation dar. Die Auswahl des Elektrodenträgerdesigns, abgestimmt auf die individuelle Anatomie der Cochlea, gewinnt dabei im Rahmen der personalisierten Cochleaimplantatversorgung an Bedeutung. Dazu kann anhand der präoperativen Bildgebung, mittels eines Tablet-basierten Softwarepakets, die Länge des Ductus cochlearis (CDL) bestimmt werden. Die Studie prüft, ob die Modalität der Bildgebung und der fachliche Hintergrund der Untersucher die CDL-Messgenauigkeit beeinflussen.

Cochleaimplantate (CI) gelten aktuell als die erfolgreichsten Neuroprothesen der Geschichte, mit alltagsrelevanten Verbesserungen des Sprachverstehens und räumlichen Hörens bei Patienten im Säuglings- bis Seniorenalter [13, 15]. Die Indikation wird zunehmend auch für Patienten mit Restgehör gestellt [9]. Der Erfolg der Cochleaimplantatversorgung ist jedoch variabel und in den individuellen Fällen schwierig vorherzusagen. Der Zeitpunkt und die Dauer der Ertaubung, das Alter bei Implantation, psychosoziale Faktoren und neurokognitive Funktionen gelten als prognostisch entscheidend, sind jedoch durch das implantierende Zentrum kaum zu beeinflussen [5, 25, 35]. Daher gewinnt die optimale Position der Elektroden in der Cochlea an Bedeutung, da sie potenziell durch das operative Vorgehen und die Auswahl des Elektrodenträgerdesigns beeinflusst werden kann [12]. Bezüglich der Elektrodenträger sind verschiedene Designs erhältlich, die kontrovers diskutiert werden. Ihnen gemeinsam ist, dass eine möglichst atraumatische Insertion angestrebt wird und die Elektroden in der Scala tympani zu liegen kommen sollten, was Vorteile im Sprachverstehen verspricht [2, 18]. Dabei gibt es die geraden „lateral wall arrays“ (LW), die an der äußeren Wand der Cochlea liegen und die peripheren Fortsätze der Spiralganglienzellen (SGZ) im Bereich des Corti-Organs stimulieren sollen, sowie die vorgeformten „modiolar hugging arrays“ (MH), die sich um den Modiolus winden, um eine möglichst kurze Distanz zur direkten Stimulation der SGZ zu haben. Bei der Insertion der MH-Elektroden wird jedoch bei erhöhtem Risiko der Translokation von der Scala tympani in die Scala vestibuli ein höheres Insertionstrauma beschrieben [39]. Dies bedeutet einen möglichen Verlust von neuronalen Elementen, was insbesondere in Hinblick auf die Erhaltung eines Restgehörs vermieden werden muss [2].

Für die LW-Elektroden wird bei rein elektrischer Stimulation eine möglichst vollständige Abdeckung der Cochlea durch eine tiefe Insertion angestrebt, um eine Stimulation von Nervenendigungen bis in die apikalen Regionen der Cochlea möglich zu machen. Dabei konnten Li et al. zeigen, dass die SGZ im Apex der Cochlea stark verdichtet vorliegen und eine optimale Frequenzauflösung im Tieftonbereich deshalb am ehesten über eine Stimulation ihrer peripheren Fortsätze im Bereich der Basilarmembran möglich scheint, da die Fortsätze dort einen größeren Abstand zueinander haben [22]. Dies würde eine Annäherung an die physiologische Orts-Frequenz-Auflösung bedeuten und könnte somit neben einem besseren Sprachverstehen in einer besseren Klangqualität resultieren [17]. Jedoch ist das erhöhte Risiko einer Translokation von der Scala tympani in die Scala vestibuli bei zu tiefer Insertion zu beachten [26].

Eine zu geringe Insertion hingegen kann zu einer Stimulation überwiegend oder sogar ausschließlich der Nervenfasern in den basalen Bereichen der Cochlea führen, was zu einer Komprimierung der Frequenzdynamik führt. Patienten erfahren dann initial aufgrund der fehlenden Übereinstimmung der Orts-Frequenz-Auflösung vermehrt eine „hochfrequente“ Klangqualität, wobei Reiss et al. dabei eine Plastizität der Hörbahn beschreiben, mit der dieses initiale Hörgefühl über die Zeit kompensiert werden kann [32]. Die volle Abdeckung soll die Notwendigkeit einer Neuorganisation der Hörbahn verringern und damit die Hörrehabilitation beschleunigen. Obwohl eine optimale Elektrodenabdeckung der Cochlea insgesamt noch nicht ausreichend erforscht ist, empfehlen Mistrik and Jolly für die LW-Elektrodenträger bei rein elektrischer Stimulation eine Abdeckung von 80 % [24].

Bei erhaltenem und stabilem Restgehör kann die Möglichkeit der elektroakustischen Stimulation (EAS) zum Einsatz kommen, wobei simultan zur elektrischen Stimulation durch das CI ein mögliches Restgehör im Tieftonbereich akustisch stimuliert wird. Hierbei kann diskutiert werden, eine kürzere Elektrode zu implantieren, um das Restgehör bestmöglich zu erhalten. Jedoch muss dazu beachtet werden, dass durch die Implantation oder über die Zeit das Restgehör verloren gehen kann und dann keine elektrische Stimulation im Tieftonbereich möglich ist.

Die Größe der Cochlea ist individuell unterschiedlich, was sich in der großen Spannweite der Länge des Ductus cochlearis, definiert von rundem Fenster bis Helicotrema, abbilden lässt [36]. Dabei sind die Referenzpunkte der Messung zu beachten, da der Unterschied der gemessenen CDL auf Höhe der lateralen Wand der Cochlea (CDL_LW_), im Vergleich zu der CDL auf Höhe des Corti-Organs (CDL_OC_) mehr als 10 % betragen kann [31]. So ergeben sich in der Synchrotron-Phasenkontrast-Bildgebung (SR-PCI), der aktuell höchstauflösenden Methode, Werte für die CDL_OC_ von 32,1 mm und CDL_LW_ von 39,0 mm [20]. Messungen anhand von Flachdetektor-Volumen- Computertomographien (fpVCT) zeigen eine CDL_OC_ von 34,63 ± 1,47 mm [27]. Die CDL_OC_ ist dabei in Zusammenhang mit der Greenwood-Funktion für die Frequenzbestimmung von zentraler Bedeutung [16]. Für die Ausmessung der CDL sind unterschiedliche Methoden bekannt. Breitspeicher et al. untersuchten die Verlässlichkeit dreier Methoden im Vergleich, eine Messung mittels 3‑D-Segmentation, die „A-Wert-Methode“ nach Escude et al. sowie die Nutzung einer otochirurgischen Planungssoftware, und kamen zu dem Ergebnis, dass die Vermessung anhand der Planungssoftware, basierend auf der sog. Elliptic-Circular -Approximation(ECA)-Gleichung, die beste Interraterreliabilität und höchste Korrelation mit den Referenzwerten ergab [6, 11, 34]. Auch Rak et al. bezeichnen in einem Review die Berechnung auf Basis der ECA-Formel als die Möglichkeit der genauesten Bestimmung der CDL, da sie Werte vergleichbar derer aus 3‑D-Rekonstruktionen liefert [31]. Nach Schurzig et al. wird die CDL-Berechnung aus der prozentualen Länge der basalen Windung ermöglicht, wobei diese mittels ECA anhand des Durchmessers (A-Wert) und der orthogonal dazu stehenden Breite (B-Wert) der basalen Windung der Cochlea geschätzt wird [34].

Die in dieser Studie verwendete Tablet-basierte Planungssoftware OTOPLAN (Fa. CAScination, Bern, Schweiz, und Fa. MED-EL, Innsbruck, Österreich) berechnet die CDL basierend auf der ECA-Methode, nachdem der Untersucher manuell Landmarken der Cochlea ausgewählt hat. Anschließend können die verfügbaren Elektrodenträger in Relation zur individuell errechneten Orts-Frequenz-Abbildung, welche anhand der Greenwood-Funktion vorausgesagt wird, dargestellt werden [16]. Die Software soll somit als einfach zu bedienende Entscheidungshilfe im klinischen Alltag eingesetzt werden. Sie wurde durch mehrere Autoren anhand von Multislice-CT (MSCT) mit der Frage nach der Intra- und Interraterreliabilität getestet, wobei sich Ergebnisse bezüglich der Messung von A‑ und B‑Werten sowie der CDL-Berechnung reliabel zeigten [7, 8, 23].

Die CDL-Messungen wurden in bisher publizierten Studien in der Regel von Neuroradiologen und Otologen durchgeführt, was in Anbetracht der komplexen Anatomie des Innenohrs nachvollziehbar ist. Die Indikation zur Implantation und die Elektrodenträgerauswahl erfolgt letztendlich durch den Operateur. Die Patientenberatung und lebenslange postoperative Patientenbetreuung erfolgt jedoch maßgeblich durch die Abteilung für Audiologie, weshalb es naheliegt, Audiologen in die Vermessung einzubeziehen. Wir stellen uns deshalb die Frage, ob der Untersucherhintergrund und damit das Maß an Erfahrungen in der Versorgung von CI-Patienten und Beurteilung von Bildgebung mittels Magnetresonanztomographie (MRT) und CT in der CDL-Bestimmung einen Effekt auf die Messergebnisse hat.

In Anlehnung an die deutsche S2k-Leitlinie zur Cochleaimplantatversorgung erhalten Patienten über 18 Jahren in der präoperativen Diagnostik eine MRT des Schädels sowie eine CT des Felsenbeins, die sich in ihrem diagnostischen Wert ergänzen [9]. Die CT ermöglicht die detaillierte Darstellung des knöchernen Felsenbeins mit Hinweisen auf Fehlbildungen, Osteolysen, die Belüftung des Mastoids, das Mittelohr mit potenziellen Besonderheiten am runden Fenster und zeigt den Verlauf des Falloppio-Kanals und des Sinus sigmoideus [38]. In Hinblick auf die Darstellung der Cochlea ist die Bestimmung der lateralen Begrenzung und die Lokalisation des runden Fensters durch den starken Kontrast des Knochens zu den bindegewebigen und flüssigkeitsgefüllten Strukturen der Cochlea eindeutig. Wir führen hierzu Flat-Panel-Volume-CT durch, wobei im Vergleich zu Multislice-CT eine höhere Bildauflösung erreicht werden kann, aus der sich durch sekundäre Rekonstruktionen potenziell eine Voxelkantenlänge von < 0,1 mm generieren lässt [29]. Dazu sind fpVCT in der postoperativen Lagekontrolle weniger artefaktanfällig [19]. Optimale Messbedingungen können sich dabei aus einer Kombination aus sekundär rekonstruierten fpVCT und der Nutzung der Software OTOPLAN ergeben [27]. Die MRT erfolgt zum Ausschluss einer retrocochleären Pathologie, der Beurteilung des Hörnervs und der Flüssigkeitsfüllung der Cochlea sowie zur Detektion bindegewebiger Obliterationen und früher Stadien der Ossifikation [38]. Die Abgrenzung des membranösen Labyrinths der Cochlea und der intracochleären Flüssigkeit zum umgebenden Knochen ist dabei weniger scharf als in der CT, weshalb für die CDL-Messungen aktuell die CT den Goldstandard darstellt. Bei Patienten unter 18 Jahren wird aufgrund der Strahlenbelastung auf eine standardmäßige CT verzichtet. Ehrmann-Müller et al. haben dazu bei Kindern unter 36 Monaten untersucht, dass der Verzicht auf eine präoperative Felsenbein-CT ein sicheres Vorgehen ohne zusätzliches perioperatives Risiko darstellt [10]. Um bei Patienten unter 18 Jahren die CDL-Messung einzig an der MRT durchzuführen und somit auch diesen Patienten eine angepasste Cochleaimplantatversorgung anbieten zu können, ist ein direkter Vergleich zwischen Messungen an MRT und CT unerlässlich.

Das Ziel dieser Studie ist es, die einzelnen und kombinierten Effekte von Bildgebungsmethode (MRT/fpVCT) und Untersucherhintergrund (Audiologe, Assistenzarzt, Oberarzt) auf die CDL-Werte zu untersuchen.

## Methoden

### Patientenkollektiv

Zehn postlingual ertaubte Patienten (2 w., 8 m.) im Alter von 29–71 Jahren (Mittelwert = 57 ± 13,9 Jahre), die sich im Zeitraum von 11/2019 bis 03/2021 in unserer Poliklinik zur Planung eines Cochleaimplantats vorstellten, wurden für die Studie analysiert. In Vorbereitung auf die Cochleaimplantatversorgung erhielten alle Patienten folgende Bildgebung nach standardisiertem Protokoll:

- MRT des Schädels (Magnetom Avanto 1,5 T, Fa. Siemens, Erlangen): CISS-Sequenz, TR(Repetitionszeit) = 5,59 ms, TE(Echozeit) = 2,3 ms, Flipwinkel 80°, Messzeit 9 min 35 s, Voxelkantenlänge 0,63 mm × 0,63 mm × 0,6 mm
- fpVCT des Felsenbeins (Axiom Artis, Fa. Siemens Healthineers AG, Erlangen): 20 s DCT; „tube voltage“: 109 kV; „rotation angle“: 198,4°; „frame angulation step“: 0,40°/f; „pulse length“: 10,0 ms

Um unnötige Bildgebungen zu vermeiden und die Vergleichbarkeit der Ergebnisse zu erhöhen, wurden Patienten mit bereits erfolgter externer Bildgebung nicht in die Studie eingeschlossen. Die Auswertung erfolgte retrospektiv, und alle Untersucher wurden verblindet bezüglich der Messungen. Die Studie wurde durch die Ethikkommission der Universität Regensburg genehmigt (Z-2020-1534-9-I).

### OTOPLAN

**Figure 1:**
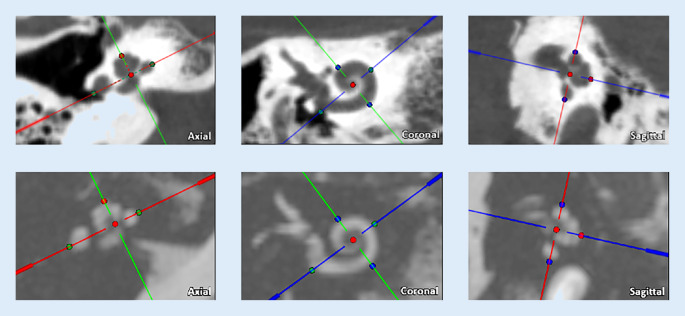

Die 20 Cochleae der 10 Probanden wurden jeweils an MRT und fpVCT ausgemessen. Dazu wurden die Datensätze in der Software OTOPLAN (Fa. CAScination, Bern, Schweiz, und Fa. MED-EL, Innsbruck, Österreich), Version 3 1.5.0, hochgeladen. Dem Algorithmus der Software folgend definierten die Untersucher nach Einstellung der schräg koronalen Ansicht der basalen Windung der Cochlea, des „cochlear views“, manuell Punkte zur Bestimmung des Durchmessers (Wert A: Strecke von rundem Fenster durch den Modiolus zur lateralen Wand) und der Breite (Wert B: senkrecht zum Durchmesser stehende Strecke zwischen den gegenüberliegenden Punkten der lateralen Wand) sowie anschließend der Höhe (Wert H: Strecke zwischen Mittelpunkt der basalen Windung und senkrecht darauf stehendem Apex; Abb. 1). In der verwendeten Version errechnete die Software mittels A‑ und B‑Werten basierend auf der ECA-Methode die CDL_LW_ [34]. Nach Alexiades et al. wurde die Formel unter Beachtung der Distanz zwischen lateraler Wand und Corti-Organ erweitert, um auf die CDL_OC_ schließen zu können [1]. Außerdem wurde der als „Hook Region“ bezeichnete Teil der Basilarmembran einbezogen, der dem Zentrum des runden Fensters vorangeht.

### Untersucher

Um den Effekt der unterschiedlichen Untersucherhintergründe auf die Messwerte zu prüfen, wurden Untersucher unterschiedlicher Berufsgruppen und damit auch mit unterschiedlichem Maß an Erfahrung in der Beurteilung bildgebender Diagnostik ausgewählt. Um das Spektrum der in die Cochleaimplantatversorgung involvierten Berufsgruppen bestmöglich abzubilden, bestanden die drei Untersucher aus einer Oberärztin der Hals-Nasen-Ohren-Heilkunde mit langjähriger Erfahrung in der Cochleaimplantat-Chirurgie (Autorin PK), einer Assistenzärztin im zweiten Jahr der Facharztweiterbildung der Hals-Nasen-Ohren-Heilkunde (Autorin LW) und einem Audiologen mit langjähriger Erfahrung in der audiologischen Betreuung von Cochleaimplantatpatienten (Autor SCM).

### Datenanalyse

Alle Analysen erfolgten in R (v. 4.0.2; [30]), das Signifikanzniveau wurde mit *p* < 0,05 festgelegt. Um den Effekt der Untersucher und der Art der Bildgebung auf die Messung der Länge des Ductus cochlearis zu analysieren, wurden lineare gemischte Modelle konstruiert, mit der CDL als abhängige Variable und zwei festen Faktoren (Bildgebung [MRT/fpVCT] und Untersucher [Audiologe/Assistenzarzt/Oberarzt]), zusammen mit einem zufälligen Faktor (Teilnehmer). Zur Konstruktion der Modelle wurde die Funktion* lme* aus dem Paket *lme4* in R genutzt [3, 30]. Die Modelle wurden unter Nutzung der ANOVA-Funktion (einfaktorielle Varianzanalyse) analysiert. Paarweise Vergleiche der signifikanten Haupteffekte und Interaktionen wurden mittels der *emmeans*-Funktion angestellt, unter Verwendung der Korrekturen für die Falscherkennungsrate und Satterthwaite-Freiheitsgraden [4, 21, 33].

## Ergebnisse

Die CT- und MRT-Bilder von 10 Patienten, entsprechend 20 Cochleae, wurden untersucht. Mittelwert und Standardabweichung der CDL_OC_-Berechnung betrugen in der fpVCT 36,69 ± 1,78 mm und in der MRT 36,81 ± 1,87 mm mit einer Spannweite von 33,05–42,61 mm. Die Tab. 1 stellt die gemessenen Mittelwerte und Standardabweichungen der Werte A (Durchmesser), B (Breite) und H (Höhe) sowie die jeweils berechnete CDL_OC_ in fpVCT und MRT für die einzelnen Untersucher dar. In der MRT zeigen sich dabei unter allen Untersuchern größer gemessene B‑Werte im Vergleich zu den Messungen in der fpVCT mit einer daraus resultierenden länger errechneten CDL_OC_. Auch die H‑Werte wurden in der MRT größer bestimmt, wobei diese nicht in die CDL_OC_-Berechnung einfließen. Die Standardabweichungen unterschieden sich jedoch nicht, es besteht keine größere Streuung unter den Messungen.

**Table 1:** 

	Audiologe		Assistenzarzt		Oberarzt	
	fpVCT	MRT	fpVCT	MRT	fpVCT	MRT
Durchmesser	9,29 ± 0,47	9,20 ± 0,43	9,32 ± 0,34	9,32 ± 0,53	9,33 ± 0,51	8,94 ± 0,45
Breite	7,02 ± 0,45	7,16 ± 0,34	6,88 ± 0,33	7,16 ± 0,46	6,98 ± 0,33	7,32 ± 0,44
Höhe	3,98 ± 0,21	4,38 ± 0,27	3,69 ± 0,25	4,37 ± 0,25	3,88 ± 0,34	4,31 ± 0,29
CDL_OC_	36,40 ± 1,51	37,26 ± 1,58	36,33 ± 1,51	37,43 ± 2,30	36,77 ± 1,75	37,47 ± 1,90

Bei einem Patienten zeigten sich im Bereich der rechten Cochlea Osteolysen sowie eine Obliteration der basalen Windung. Dieser Befund machte die Ausmessung nach Aussage der Untersucher subjektiv schwieriger, da die Markierung der lateralen Wand und des runden Fensters der Cochlea aufgrund der wolkigen Veränderungen nicht eindeutig festgelegt werden konnte. Dennoch zeigten die Messwerte dieses Patienten mit einer mittleren CDL_OC_ in der CT von 35,6 mm und in der MRT von 35,5 mm keinen nennenswerten Unterschied.

**Figure 2:**
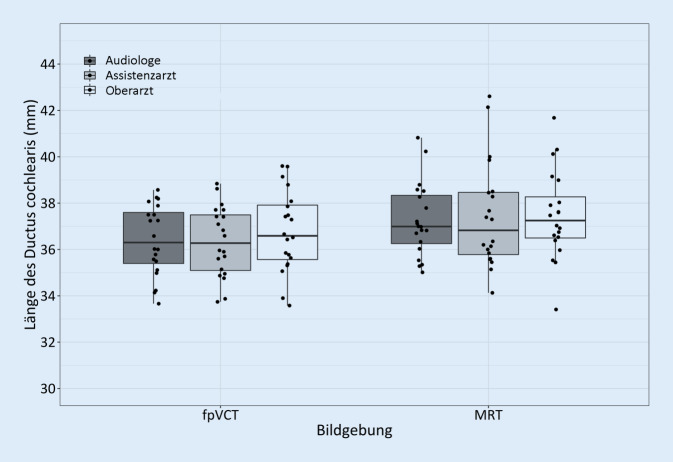

**Figure 3:**
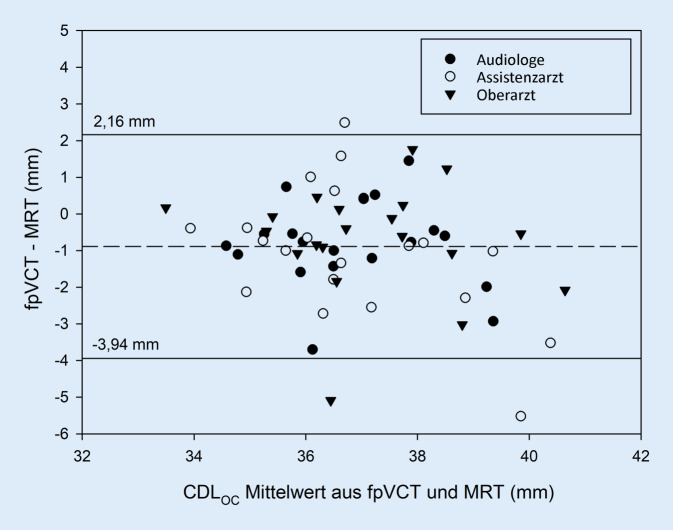

**Figure 4:**
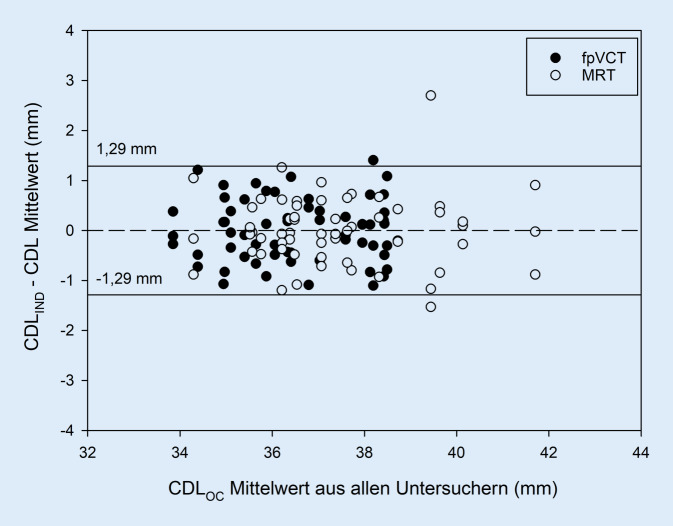

Die Streuung der absoluten CDL-Messwerte der drei Untersucher für MRT und fpVCT ist als Box-Whisker-Plot in Abb. 2 dargestellt. Im Bland-Altman-Diagramm zum Vergleich der beiden Bildgebungsmodalitäten in Abb. 3 zeigen sich wenige inakzeptable Abweichungen (3 von 60). Auch in Abb. 4 im Bland-Altman-Diagramm zum Vergleich der drei Untersucher fallen wenige klinisch inakzeptable Abweichungen auf (3 von 120).

Die einfaktorielle Varianzanalyse mittels ANOVA in Bezug auf die Bildgebungsmodalität zeigte einen signifikanten Haupteffekt: F (1, 105) = 20,70; *p* = 0,000015. Dabei waren die vermessenen Werte der CDL in der MRT größer als in der CT (*M* Differenz = 0,89 mm; 95%-Konfidenzintervall 0,50–1,28). Unsere Analyse der Untersucher zeigte keinen signifikanten Effekt: F (2, 105) = 0,84; *p* = 0,437. Auch die Untersucher × Bildgebung-Interaktion zeigte keine Signifikanz: F (2, 105) = 0,342; *p* = 0,711. Die Unterschiede zwischen den Messungen der verschiedenen Untersucher waren für beide Bildgebungsmodalitäten klein und weder statistisch noch klinisch signifikant (*M* Differenz < 0,5 mm).

## Diskussion

Das Ziel dieser Studie war es, mögliche Unterschiede in den Messergebnissen der CDL-Bestimmung mit einer Tablet-basierten Planungssoftware in Abhängigkeit von der Bildgebungsmodalität (fpVCT, MRT) und des Untersucherhintergrunds (Oberärztin, Assistenzärztin, Audiologe) darzulegen.

Unsere Messwerte der CDL_OC_ in der fpVCT mit einem Mittelwert von 36,7 ± 1,8 mm sind vergleichbar mit den Werten publizierter Studien. Müller-Graff et al. beschreiben in nicht implantatversorgten Cochleae in der fpVCT einen Mittelwert der CDL_OC_ von 34,63 ± 1,47 mm, wobei hier die OTOPLAN, Version 2, verwendet wird. In dieser wird die „Hook Region“ mit 1,58 mm statt wie in Version 3 mit 2,5 mm einberechnet, was den Messunterschied zum Teil erklärt. Bei Breitsprecher et al. ergibt sich in der Ausmessung in der hochauflösenden CT mit der verwendeten Planungssoftware eine mittlere CDL_OC_ von 37,0 ± 1,8 mm, hier wird die CDL_OC_ durch Multiplikation der CDL_LW_ mit 0,9 bestimmt [6]. Spiegel et al. beschreiben einen Mittelwert von 36,2 ± 1,8 mm in der MSCT, wobei am ehesten die CDL_LW_ angegeben wird [36].

In der Vermessung der CDL an der MRT beschreiben Taeger et al. mittels Elliptic Circular Approximation einen Mittelwert von 36,59 ± 2,64 mm [37]. Obwohl in unserer Studie die Erweiterung der ECA-Formel zur Berechnung der CDL_OC_ genutzt wird, zeigte sich ein vergleichbarer Mittelwert an der MRT von 36,81 ± 1,87 mm. Diese Vergleiche unterstreichen die notwendige Sensibilität zur Beachtung der verwendeten Formeln in der CDL-Berechnung, da diese die Ergebnisse beeinflussen können [6]. Dabei muss als Limitation unserer Studie beachtet werden, dass aufgrund der kleinen Patientenzahl von 10 kein Rückschluss auf eine gesamte Population zugelassen werden kann und sich daraus dennoch Unterschiede zu vergleichbaren Studien ergeben können.

Es wurden von jedem Patienten jeweils beide Cochleae vermessen. Die CDL beider Cochleae innerhalb eines Patienten korreliert signifikant, sodass die Streuung zwischen den Patienten unterschätzt wird. Linear gemischte Modelle wurden deshalb verwendet, um den Einfluss der Korrelation zwischen den Ohren zu kontrollieren. Die Interrater- und Intraraterreliabilität der Messungen mithilfe der Software OTOPLAN, basierend auf der ECA-Methode, wurden für verschiedene Untersucher in mehreren Studien als exzellent bezeichnet [6, 7, 23]. Da die Reliabilität in unserer Studie nicht erneut untersucht wurde, stellt die in der Literatur gezeigte Verlässlichkeit dieser Methoden die Grundlage für die weitere Berechnung der Effekte des Untersucherhintergrunds und der Bildgebungsmodalität auf die CDL-Messungen dar.

In den Analysen bezüglich des Untersucherhintergrunds konnten statistisch keine signifikanten Effekte aufgezeigt werden. Die Darstellung in Abb. 4 zeigt eine hohe Zuverlässigkeit der Messungen zwischen den drei Untersuchern, die in MRT und fpVCT vergleichbar ist. In der MRT fällt insgesamt eine Tendenz zu größeren CDL_OC_-Messungen auf, wobei sich auch in den berechneten längeren Mittelwerten keine höhere Streuung zwischen den Untersuchern zeigt. Demnach kann die Software in der Schätzung der CDL auch verlässlich von Personal ohne langjährige Erfahrung in der Interpretation von bildgebender Diagnostik bedient werden. Unsere Resultate suggerieren, dass auch Fachpersonal ohne ärztliche Ausbildung in die Auswertung einbezogen werden sollte. So könnten beispielsweise betreuende Audiologen die CDL-Messungen vornehmen, anhand derer dann implantierende Chirurgen die Elektrodenauswahl treffen können. Dadurch können Planungsabläufe optimiert und beschleunigt werden. Weitere Untersuchungen wären notwendig, um zu identifizieren, welche Erfahrung oder Mindestqualifikationen notwendig sind, um die CDL akkurat zu bestimmen.

Die Modalität der Bildgebung, wobei fpVCT und MRT untersucht wurden, zeigte einen statistisch signifikanten Einfluss auf die Ausmessung der CDL_OC_. Die CDL_OC_-Schätzungen anhand der MRT waren im Vergleich zur fpVCT im Mittel um 0,89 mm größer. Die Gefahr der Überschätzung der CDL_OC_ bei alleinigen Messungen an der MRT sollte daher beachtet werden. Auch bei der Betrachtung der einzelnen Messwerte A, B und H fällt auf, dass unter allen Untersuchern die B‑ und H‑Werte in der MRT im Vergleich zur fpVCT am ehesten überschätzt wurden. Ein Erklärungsversuch zielt auf das fehlende Knochensignal in der CISS-Sequenz der MRT ab, zusammen mit der im Vergleich geringeren Auflösung resultiert ein eher fließender Übergang im Bereich der lateralen Wand der Cochlea, wodurch die Markierungen systematisch weiter außen gesetzt wurden. Die A‑Werte wurden in der MRT dabei annähernd gleich oder sogar kleiner als in der fpVCT bestimmt, obwohl in Anbetracht der ansonsten größer gemessenen Werte auch hier eine Überschätzung zu erwarten wäre. Die in der MRT schwierigere Lokalisation des runden Fensters könnte dafür ursächlich sein. Die Abb. 3 zeigt bei nur wenigen inakzeptablen Abweichungen dennoch eine hohe Vergleichbarkeit der Messungen anhand der fpVCT und MRT unter allen Untersuchern. Es sollte jedoch beachtet werden, dass unter diesen Abweichungen die gemessenen Differenzen zwischen fpVCT und MRT im Einzelfall, wie hier von bis zu 5,5 mm, durchaus eine klinische Bedeutung für die Auswahl einer angepassten Elektrodenträgerlänge haben. Da auch Messfehler zu solchen Ausreißern führen können, ist aufgrund dieser berechneten Werte zu empfehlen, die Messungen jeweils durch einen ebenso geschulten Mitarbeiter kontrollieren zu lassen. Ist der Unterschied tatsächlich relevant, müssen die Messungen kritisch überprüft werden. Auch in vergleichbaren Studien wurden Messunterschiede beobachtet. Taeger et al. beschrieben Unterschiede in der CDL-Messung von CT und MRT von 0,65 mm, wobei die CDL in den MRT systematisch eher unterschätzt und der Unterschied als statistisch nicht signifikant gewertet wurde. George-Jones et al. zeigten eine kürzer gemessene mittlere CDL in der MRT (31,9 ± 2,4 mm) als in der CT (32,7 ± 2,0 mm) [14]. Nash et al. verglichen die CDL in der CT und MRT anhand der A‑Wert-Methode und stellten einen Unterschied von 0,96 mm fest [28]. Trotz der gemessenen Unterschiede wurde die alleinige CDL-Messung an der MRT in allen hier aufgeführten Publikationen als sicheres Verfahren gewertet. Es stellt sich die Frage, ob unser statistisch signifikanter Unterschied mit einer mittleren Differenz zwischen fpVCT und MRT von 0,89 mm auch eine klinische Relevanz zeigt. Theoretisch ist bei allein an der MRT zu lang geschätzter CDL_OC_ als systematischer Fehler die Auswahl einer zu langen Elektrode möglich, was eine zu tiefe Insertion zur Folge haben kann. Dies kann ein höheres intracochleäres Trauma mit einer höheren Rate an Translokationen bedingen [26]. Eine zu lange Elektrode kann potenziell nicht vollständig in die Cochlea eingeführt werden, wobei außenliegende Kontakte nicht genutzt werden können. Sollte der Fall eintreten, dass nach einer Ausmessung alleinig an der MRT die 80 %ige Abdeckung der Cochlea nach Empfehlung von Mistrik und Jolly zwischen zwei der angebotenen Größen liegen, kann diskutiert werden, ob eine Entscheidung zugunsten der kürzeren Elektrode gefällt werden sollte, um die genannten Risiken bestmöglich zu vermeiden [24]. Da die optimale Abdeckung der Cochlea jedoch noch nicht abschließend untersucht ist, kann dazu keine generelle Empfehlung ausgesprochen werden. Die Differenz von 0,89 mm bedeutet nach Berechnung anhand der durchschnittlichen CDL_OC_ und einem Flex-28-Elektrodenträger jedoch nur einen Unterschied in der apikalen Elektrode von ca. 50 Hz. Der gemessene Unterschied wird daher als statistisch signifikant, klinisch jedoch nicht relevant beurteilt. Eine alleinige Ausmessung an MRT-Bildern ist deshalb möglich, um auch Patienten unter 18 Jahren eine Vermessung anzubieten.

Unter der Annahme, dass eine ohrspezifisch ausgewählte Elektrodenträgerlänge klinisch signifikante und langanhaltende Vorteile für CI-Nutzer liefert, bestätigt die Spannweite unserer CDL-Messungen von 33,05–42,61 mm, dass die CDL individuell zu unterschiedlich ist, um eine einzige Elektrodenträgerlänge als passend zu bezeichnen. Dabei ist es gerade für die bestimmten Patienten mit kleinerer oder größerer Cochlea wichtig, mit entsprechenden Elektrodenträgern auf die spezielle Anatomie reagieren zu können. Die Studie hat gezeigt, dass die Vermessung der CDL eine im klinischen Alltag einfach anzuwendende Methode ist. Damit ist die Grundlage für weitere Studien bezüglich einer personalisierten Cochleaimplantatversorgung und eines Einflusses der angepassten Elektrodenlänge auf das Endergebnis des offenen Sprachverstehens geschaffen.

## Fazit für die Praxis

- Die CDL ist individuell unterschiedlich, weshalb deren Bestimmung eine wichtige Voraussetzung für eine personalisierte Implantatauswahl ist.
- Die Methode der Bildgebung kann die CDL-Werte statistisch signifikant beeinflussen, auf eine klinische Relevanz kann anhand unserer Berechnungen nicht geschlossen werden.
- Die Vermessung alleinig an MRT ist ein sicheres Verfahren.
- Geschultes Fachpersonal sollte in die CDL-Messung einbezogen werden, um Abläufe bezüglich der Implantatauswahl durch implantierende Chirurgen/-innen zu beschleunigen.
